# Revealing the Adsorption Mechanisms of Methanol on Lithium-Doped Porous Carbon through Experimental and Theoretical Calculations

**DOI:** 10.3390/nano13182564

**Published:** 2023-09-15

**Authors:** Yiting Luo, Muaoer Fang, Hanqing Wang, Xiangrong Dai, Rongkui Su, Xiancheng Ma

**Affiliations:** 1 Hunan First Normal University, Changsha 410114, China; 2 College of Mechanical and Electrical Engineering, Central South University of Forestry and Technology, Changsha 410004, China; 3 College of Environmental Science and Engineering, Central South University of Forestry and Technology, Changsha 410004, China; 4 PowerChina Zhongnan Engineering Corporation Limited, Changsha 410004, China

**Keywords:** porous carbon, methanol adsorption, lithium doping, pore structure, adsorption mechanism

## Abstract

Previous reports have shown that it is difficult to improve the methanol adsorption performance of nitrogen and oxygen groups due to their low polarity. Here, we first prepared porous carbon with a high specific surface area and large pore volume using benzimidazole as a carbon precursor and KOH as an activating agent. Then, we improved the surface polarity of the porous carbon by doping with Lithium (Li) to enhance the methanol adsorption performance. The results showed that the methanol adsorption capacity of Li-doped porous carbon reached 35.4 mmol g^−1^, which increased by 57% compared to undoped porous carbon. Molecular simulation results showed that Li doping not only improved the methanol adsorption performance at low pressure, but also at relatively high pressure. This is mainly because Li-modified porous carbon has higher surface polarity than nitrogen and oxygen-modified surfaces, which can generate stronger electrostatic interactions. Furthermore, through density functional theory (DFT) calculations, we determined the adsorption energy, adsorption distance, and charge transfer between Li atom and methanol. Our results demonstrate that Li doping enhances the adsorption energy, reduces the adsorption distance, and increases the charge transfer in porous carbon. The mechanism of methanol adsorption by Li groups was revealed through experimental and theoretical calculations, providing a theoretical basis for the design and preparation of methanol adsorbents.

## 1. Introduction

The excessive release of volatile organic compounds (VOCs) poses significant hazards to both human health and the environment [1,2]. These compounds, including benzene, formaldehyde, and xylene, readily evaporate at room temperature. VOCs react with nitrogen oxides in the air to form ozone, which can irritate the respiratory system. Prolonged exposure to VOCs can lead to respiratory diseases and immune system abnormalities [3,4]. Methanol, consisting of CH_3_ and OH groups, is a significant industrial raw material and reagent. It is one of the most common VOCs gases in the atmosphere, and contributes to the formation of photochemical smog and poses a threat to the human nervous system [5,6]. Consequently, it is crucial to investigate efficient methanol adsorbents to enhance their extensive utilization in industrial processes. 

Adsorption technology is considered one of the most mature and effective methanol control technologies [7,8]. The key to adsorption technology lies in the development of high-performance adsorbents. Commonly used adsorbents, such as porous carbon [9,10,11], metal-organic frameworks (MOFs) [12,13], mesoporous silica [14], and zeolites [15], have been used as adsorbents to reduce VOCs in polluted media. Porous carbon, with advantages such as easy surface modification, high specific surface area, tunable pore structure, and low cost, is considered to be a promising adsorbent [9,16,17]. For porous carbon, surface chemistry and pore structure are two important factors determining its methanol adsorption performance [18,19,20]. Previous studies have shown that nitrogen and oxygen doping has been found to enhance methanol adsorption at low pressure, while the total pore volume determines the adsorption performance at high pressure. For example, Ma et al. [10] conducted experiments on biomass-based porous carbons to evaluate their methanol adsorption performance. The results showed that oxygen groups could increase the adsorption capacity of methanol at relatively low pressures, while at relatively high pressures the adsorption capacity of methanol varied consistently with the total pore volume of the porous carbon, without a significant relationship with the oxygen groups. This is mainly because oxygen groups have low polarity, making it difficult to improve the methanol adsorption performance at relatively high pressure. Recently, alkali metal doping has been found to provide higher surface polarity compared to nitrogen, oxygen, and sulfur doping, improving gas adsorption [21,22]. For example, Ma et al. [22] demonstrated that the incorporation of alkali metals into porous carbon significantly enhances its CO_2_ absorption capacity, reaching values of 8.43–12.46 mmol g^−1^. This represents a 3–4-fold increase compared to undoped porous carbon and a 2–3-fold increase compared to nitrogen/oxygen-doped porous carbon. However, to date, there is a lack of research investigating the potential of alkali metal-doped porous carbon for methanol adsorption. 

Benzimidazole is a cost-effective and commercially viable nitrogen-rich organic compound, which serves as a suitable single-source precursor for the synthesis of heteroatom-doped porous carbons. For instance, Wu et al. [23] prepared porous carbon materials using benzimidazole as a carbon source, which exhibited a specific surface area of 3000 m^2^ g^−1^, a total pore volume of 2.8 cm^3^ g^−1^, an oxygen content of 5.6 at%, and a nitrogen content of 1.7 at%. In this study, we first prepared porous carbon with high specific surface area and pore volume using benzimidazole as a carbon precursor and KOH as an activating agent. Then, Li doping was employed to enhance the surface polarity of the carbon-based materials and improve the methanol adsorption performance. Finally, the isothermal adsorption curve of methanol on the Li-doped slit pore model was calculated using grand canonical Monte Carlo (GCMC) simulation, and the impact of Li groups and pore size on the methanol adsorption performance was analyzed. Density functional theory (DFT) was used to calculate the adsorption energy, electron transfer and adsorption distance between Li group and methanol molecules. The synergistic adsorption of methanol by Li doping and pore size was studied based on the results of GCMC and DFT calculations, and the adsorption mechanism was revealed.

## 2. Experimental Section

### 2.1. Material Synthesis

Preparation of Porous Carbon: Using benzimidazole as carbon precursor and KOH as activator, porous carbons with well-developed pores were prepared with the chemical activation method. The specific procedure is as follows: Firstly, KOH and benzimidazole were mixed in a ratio of 3:1 and thoroughly ground. The mixture was then transferred into a quartz boat and placed in a tube furnace. The composite material was placed in a tubular furnace and purged with nitrogen gas for 30 min to remove the air inside the furnace. Then, the temperature was increased at a rate of 5 °C min^−1^ under a nitrogen gas atmosphere until reaching 800 °C, and held for 1 h. After that, the temperature was decreased at a rate of 5 °C min^−1^ until reaching room temperature. The obtained product was placed in a 5 wt% HCl solution and stirred with a magnetic stirrer for 1 h to remove excess potassium salts. Finally, the sample was washed with deionized water and filtered, followed by drying in a 120 °C drying oven for 6 h. The resulting sample was denoted as PC.

Preparation of Li-doped porous carbon: A solution of LiOH (20 mg mL^−1^) was taken in volumes of 5 mL, 10 mL, and 25 mL, and mixed thoroughly with 0.1 g of PC. After drying, the mixture was subjected to a second carbonization process in a tube furnace. The temperature was gradually elevated at a rate of 5 degrees Celsius per minute until it reached the pinnacle of 600 degrees Celsius, and then maintained for 1 h. Subsequently, the temperature was lowered to room temperature. The resulting samples were washed in water with a magnetic stirrer at room temperature for 10 h, followed by drying at 105 °C for 8 h. These samples were named LiPC-1 (5 mL), LiPC-2 (10 mL), and LiPC-3 (25 mL) according to the volume of LiOH solution used.

### 2.2. Material Characterization and Computational Methodology

The Supplementary Information (Text S1) provides detailed information on the characterization of materials and computational methodology. The L-J potential parameters and atomic partial charges of the gas molecule are shown in Table 1.

## 3. Results and Discussion

### 3.1. Chemical and Structural Properties of Porous Carbon

**Figure 1:**
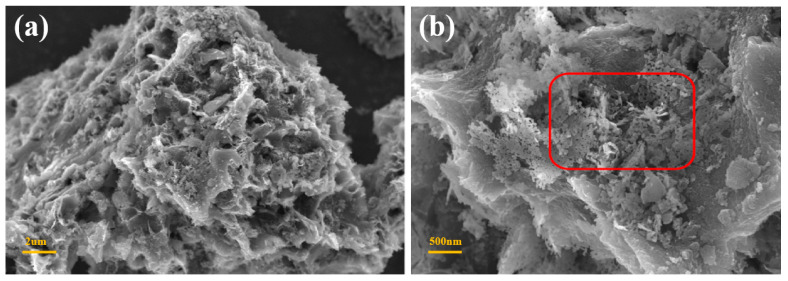
SEM images of LiPC-3, (**a**) 2 μm and (**b**) 500 nm.

**Figure 2:**
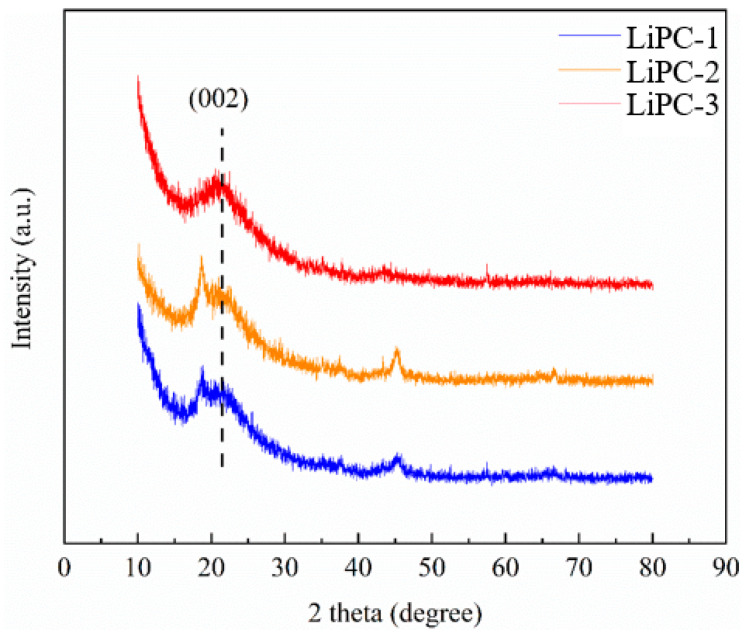
XRD pattern of Li-doped porous carbon.

The morphology of the LiPC-3 was presented using SEM images. Figure 1a displays the surface of the LiPC-3 with honeycomb-like features when the magnification scale was set to 2 μm. Figure 1b shows the randomly distributed sheet-like disordered microstructures of LiPC-3 when the magnification scale was set to 500 nm. X-ray diffraction analysis was performed on the LiPC-1, LiPC-2, and LiPC-3 samples, as shown in Figure 2. It can be observed that the common feature for different amounts of alkali metal Li doping is the broad peak at 2θ = 23°, which represents the (002) diffraction plane and indicates a partial graphitization degree of the porous carbon [24]. Additionally, the peaks of Li derivatives in the XRD pattern were not very prominent, indicating a good dispersion of Li in the porous carbon.

**Figure 3:**
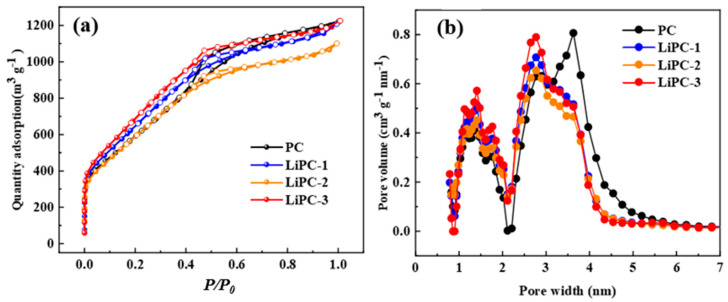
(**a**) N_2_ sorption–desorption isotherms and (**b**) pore size distributions calculated with NLDFT of porous carbon.

Figure 3 displays the N_2_ adsorption–desorption isotherms of all samples at 77 K. The N_2_ adsorption isotherm of the PC sample sharply increased with a relative pressure of P/P_0_ = 0.01, indicating the presence of micropores. It is worth noting that the isotherm of PC shows an increasing N_2_ adsorption with increasing relative pressure in the range of P/P_0_ = 0.01–0.5, along with a small hysteresis loop, indicating the presence of mesopores. After Li doping, there was no significant change in the N_2_ adsorption isotherm. As shown in Figure 3b, the pore size distribution mainly fell within two similar ranges: 1–2 nm and 2–5 nm. With the introduction of Li, the peak at 1–2 nm increased and the peak at 2–5 nm shifted to the right, indicating that Li doping could enhance the micropore volume and reduce the size of narrow mesopores in the porous carbon. As shown in Table 2, PC exhibited a high specific surface area and total pore volume of 2268 m^2^ g^−1^ and 1.716 cm^3^ g^−1^, respectively. The specific surface area of Li-doped porous carbon increased from 2268 m^2^ g^−1^ to 2678 m^2^ g^−1^, representing a 14% increase. However, the total pore volume decreased from 1.716 cm^3^ g^−1^ to 1.688 cm^3^ g^−1^. This is mainly due to the fact that LiPC-3 showed the highest micropore volume of 0.535 cm^3^ g^−1^, which was a 32% increase compared to PC. These results were mainly attributed to the activation of porous carbon by LiOH, leading to an increase in micropore volume, while the activation or pyrolysis process caused the collapse of mesopores, resulting in a decrease in mesopores.

**Figure 4:**
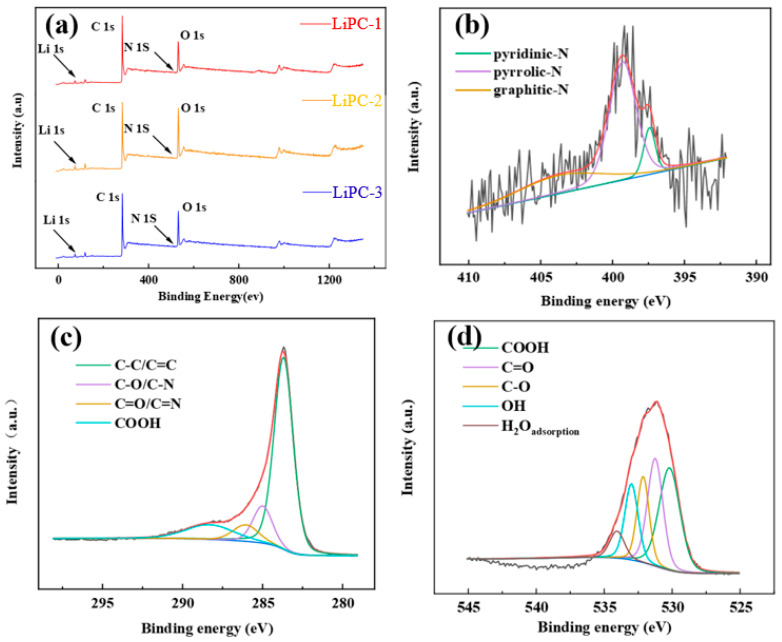
XPS spectra of the LiPC-3: (**a**) survey spectrum, (**b**) N 1s, (**c**) C 1s, and (**d**) O 1s.

The types and quantities of elements in the samples were measured using X-ray photoelectron spectroscopy (XPS). The spectrum of LiPC-3 only showed four main peaks, corresponding to C 1s, N 1s, O 1s and Li 1s (Figure 4a). As shown in Figure 4b, the N1s peak was divided into pyridinic-N (398.4 ± 0.2), pyrrolic-N (399.8 ± 0.2), and graphitic-N (400.7 ± 0.2). Pyrrolic-N was the main nitrogen functional group in all samples [25,26]. As shown in Figure 4c, the C 1s peak was divided into C-C/C=C (286.2 eV), C-O/C-N (287.4 eV), and C=O/C=N (289.0 eV) [27]. Figure 3d shows the O 1s peak divided into five peaks at 532.1 eV (COOH), 531.9 eV (C=O), 532.8 eV (C-O), 533.4 eV (OH), and 535 eV (H_2_O adsorption) [8,28]. With the increase in Li proportion, the nitrogen content decreased while the oxygen content increased (Table 2). 

### 3.2. Methanol Adsorption

**Figure 5:**
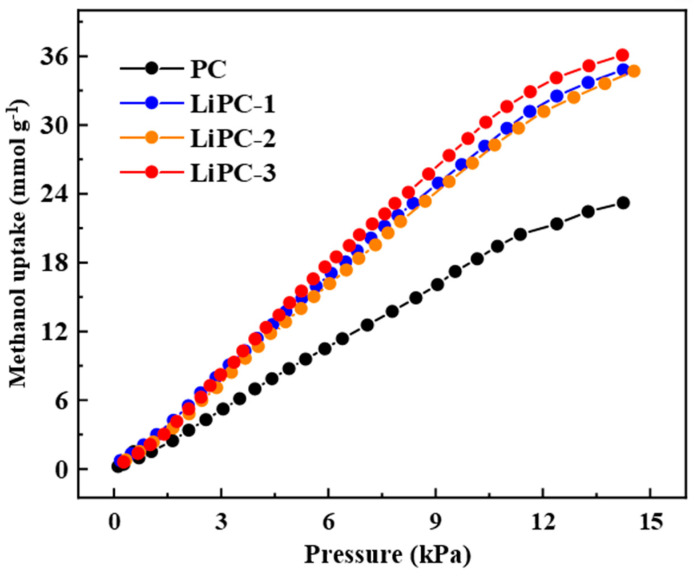
The adsorption isotherms of methanol on porous carbon at 25 °C.

The excellent pore structure and high surface area make high-performance porous carbon a promising adsorbent material. As shown in Figure 5, it can be seen that PC exhibits excellent methanol adsorption performance, with an adsorption capacity of 22.5 mmol g^−1^. In order to further increase the methanol adsorption performance, we doped Li to increase the surface polarity of the porous carbon and improve the affinity between the porous carbon and methanol. Li doping increased the methanol adsorption performance of the porous carbon. For example, LiPC-3 showed the highest methanol adsorption performance, with a capacity of 35.4 mmol g^−1^. Compared to PC, the methanol adsorption amount of LiPC-3 increased by 57%. Previous works in the literature have shown that the methanol adsorption capacity of porous carbon is mainly determined by the specific surface area and total pore volume [10,29]. The specific surface area of LiPC-3 increased by 18% compared to PC, while the total pore volume decreased. Therefore, the highest methanol adsorption capacity of LiPC-3 was mainly due to the introduction of Li into the porous carbon, which increased the surface polarity and improved the affinity between the porous carbon and methanol. Moreover, Li-doped porous carbon exhibited a superior adsorption capacity for methanol compared to other adsorbents, including porous carbons [29,30], MOFs [8,31,32,33], and metal oxide/carbon composites [34] (Table 3). This advantageous adsorption capability holds significant promise for future environmental applications.

### 3.3. Methanol Adsorption Mechanism

**Figure 6:**
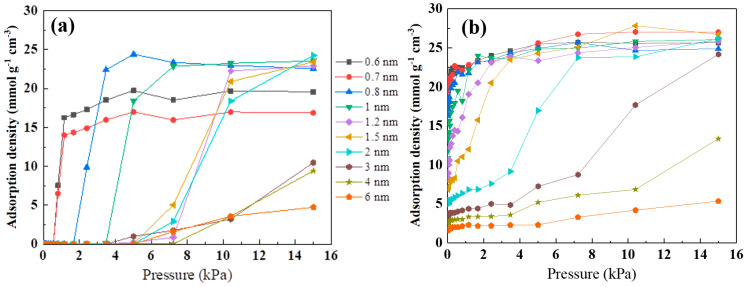
Adsorption density of the slit pore model (**a**) and Li-doped slit pore model (**b**) on methanol.

The effect of pore size and nitrogen/oxygen groups on methanol adsorption at 25 °C, as simulated by GCMC, is showed in Figure 6. As shown in Figure 6a, the methanol adsorption isotherm was similar to the water adsorption isotherm on porous carbon [40]. At 15 kPa, compared to the pore size range of 0.8–2 nm, there was a slight decrease in methanol adsorption in the pore size range of 0.6–0.7 nm. This was mainly because methanol molecules adsorbed as a monolayer in the 0.6–0.7 nm slit pore model, while there was multilayer filling adsorption in the 0.8–2 nm range. When the pore size exceeded 2 nm, the methanol adsorption sharply decreased at 15 kPa. It is worth noting that the adsorption capacity of the 6 nm pore size can be neglected compared to the 0.6 nm pore size. This could be attributed to inadequate methanol–methanol interactions in the slit pore model, which cannot support the adsorption of methanol molecules in wider pores [10,41]. To gain a better understanding of the impact of the slit pore model on methanol adsorption at low pressures, a threshold pressure was introduced. This threshold pressure represents the pressure at which the adsorbate begins to be adsorbed by the adsorbent. As the pore size increased from 0.7 nm to 6 nm, the threshold pressure increased from 0.8 kPa to 5 kPa. Additionally, the saturated adsorption capacities at 1 kPa for pores of 0.6 nm and 0.7 nm were 16 mmol g^−1^ and 14 mmol g^−1^, respectively. For pores of 0.8–2 nm, the threshold pressure to reach the saturated adsorption capacity was 3–10 kPa. However, for pores of 3–6 nm, the saturated adsorption capacity was not reached even at 15 kPa. This indicates that smaller pore sizes are advantageous for adsorbing methanol at low pressures. As shown in Figure 6b, Li doping can reduce the threshold pressure of methanol. This is primarily attributed to the increased polarity of the carbon framework surface due to the doping of Li atoms, which enables strong interactions with the OH groups in methanol. At 15 kPa, compared to pure graphite (2 nm), the pore size for filling adsorption in the slit pore model increased to 3 nm after Li doping, and the adsorption capacity in the 4–6 nm pores was also improved. In addition, at 15 kPa, the methanol adsorption capacity was also increased by 10–20%. Therefore, Li doping not only improved the adsorption performance of methanol at low pressures, but also at relatively high pressures, which was different from the results of nitrogen- and oxygen-doped porous carbons in previous works in the literature.

**Figure 7:**
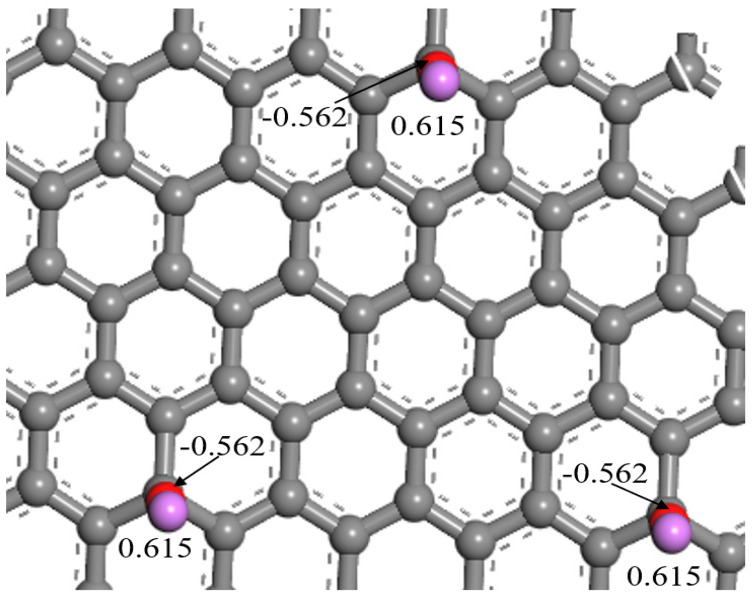
Partial charge distribution of Li-doped slit pore model surface atoms.

To better explain the electrostatic interactions between Li and methanol molecules, the charge distribution of atoms on the graphene sheet doped with Li was calculated using the Mulliken charge method. As shown in Figure 7, the Li atom in the Li-doped graphene sheet carried a strong positive charge of 0.615 *e*, while the oxygen atom connected to the Li atom carried a strong negative charge of −0.562 *e*. Therefore, Li doping led to a strong electronegativity/electropositivity on the carbon surface, which could provide effective adsorption sites for methanol and enhance its adsorption performance.

**Figure 8:**
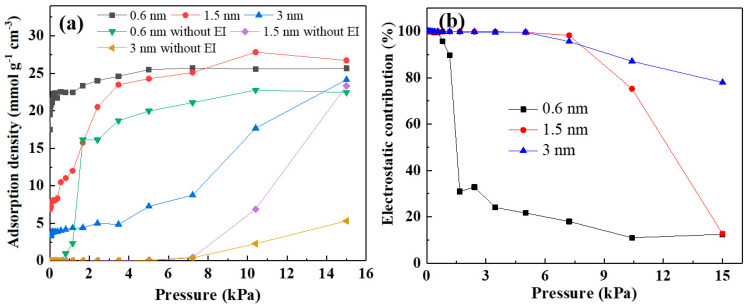
(**a**) Adsorption density of Li-doped slit pore model with EI and without EI; (**b**) electrostatic contribution of Li-doped slit pore model on methanol.

Furthermore, GCMC simulations were performed with and without electrostatic interactions between the carbon framework and methanol molecules. The adsorption capacity of porous carbon with and without electrostatic interactions for methanol is shown in Figure 8a. As shown in Figure 8, electrostatic interactions could lower the threshold pressure for methanol adsorption in the slit pore model, thereby providing a larger adsorption capacity at relatively low pressures. At relatively high pressures, electrostatic interactions could also increase the adsorption performance of methanol, increasing it by about 15% in the slit pores around 0.6 and 1.5 nm. In the 3 nm slit pore, the electrostatic effect generated by the Li-doped slit hole model increased the adsorption capacity of methanol by 357% compared with the undoped one. Additionally, we examined the influence of Li-doped porous carbon on methanol adsorption properties by estimating the electrostatic contribution using Equation (1).

(1) $$\mathrm{Electrostatic}\;\mathrm{contribution}=\frac{S_{\mathrm{with}}-S_{\mathrm{without}}}{S_{\mathrm{with}}}\times 100\%$$

The adsorption amounts of methanol molecules on Li-doped graphite sheets with and without electrostatic interactions were denoted as *S*_with_ and *S*_without_, respectively. The electrostatic contribution of graphite sheets to methanol absorption at 25 °C is shown in Figure 6b. The doping of Li enhanced the electrostatic contribution of the 0.6 nm pore to 99% at 0.5 kPa, which decreased to 30% as the pressure increased to 1 kPa, and then gradually decreased to 10% at 15 kPa. Additionally, the introduction of Li atoms resulted in electrostatic contributions of over 95% for the 1.5 nm and 3 nm pores at pressures ranging from 0 to 7 kPa. As the adsorption pressure increased to 15 kPa, the electrostatic contributions of the 1.5 nm and 3 nm pores decreased to 10% and 75%, respectively. This indicates that electrostatic interactions played a more significant role in methanol absorption at low pressures compared to high pressures. In this process, alkali metals exhibited stronger electrostatic interactions due to their higher electron accepting/donating density, thereby enhancing the capture of methanol. Furthermore, the electrostatic interactions of methanol adsorption were stronger in the slit pore model as the pore size increased. For example, the electrostatic contribution of methanol adsorption decreased to 30% at 1.5 kPa for a pore size of 0.6 nm, while the electrostatic contributions of pore sizes 1.5 nm and 3 nm still remained at 99%. Additionally, at 15 kPa, the electrostatic contributions of pore sizes 0.6 nm and 1.5 nm were only around 13%, while the electrostatic contribution of the 3 nm pore size remained at 80%.

**Figure 9:**
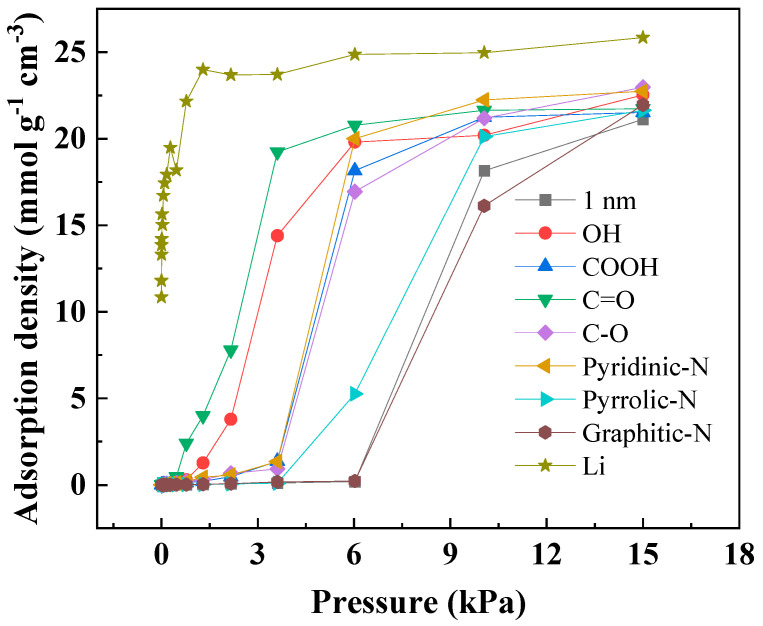
Adsorption density of O-, N-, and Li-doped slit pore model on methanol.

The isotherm effects of lithium-, nitrogen-, and oxygen-doped narrow pores on methanol adsorption are illustrated in Figure 9. At relatively low pressure, nitrogen and oxygen doping could reduce the threshold pressure for methanol adsorption and increase the adsorption capacity of methanol, while the saturated adsorption capacity of methanol showed no significant difference. The adsorption capacity of methanol at 15 kPa ranged from 21.9 to 22.9 mmol g^−1^, which was 3.8–8.5% higher than that of undoped methanol (21.1 mmol g^−1^). This indicates that nitrogen and oxygen groups have limited influence on the adsorption capacity of methanol under relatively high pressure, but can enhance the adsorption capacity of methanol under relatively low pressure. The lithium-doped slit pores reached saturation at 1 kPa, while the saturation pressures for nitrogen and oxygen doping were both above 2.5 kPa, which mainly meant that the slit pore models produced by lithium doping had stronger surface polarity than those of doped nitrogen and oxygen. Additionally, the saturated adsorption amount of lithium-doped narrow pores increased by approximately 10–25% compared to nitrogen and oxygen doping. This was mainly due to the higher surface polarity of the lithium-modified porous carbon compared to nitrogen and oxygen modifications.

**Figure 10:**
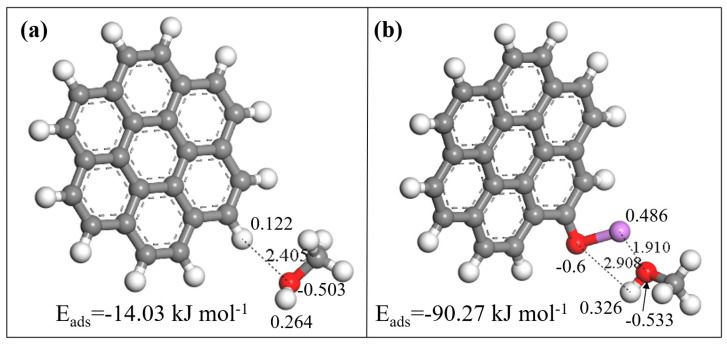
Adsorption energies of perfect graphene (**a**) and Li-doped graphene (**b**).

The optimal adsorption sites of methanol on graphite are illustrated in Figure 10. As shown in Figure 10, the adsorption energy of methanol on graphite was only −14.03 kJ mol^−1^, while the adsorption energy of methanol on lithium-modified graphite reached −90.27 kJ mol^−1^, significantly higher than that of the unmodified graphite. We calculated the adsorption distance between functional groups and methanol molecules and found that the adsorption distance between lithium atoms in porous carbon and oxygen atoms in methanol is 1.91 Å, lower than the distance between hydrogen in graphite and oxygen in methanol (2.41 Å). In addition, the charge transfer during the adsorption process was also calculated. Hydrogen atoms in graphite transferred 0.03 electrons to oxygen atoms in methanol, while lithium-doped graphite transferred 0.33 electrons to oxygen atoms in methanol, significantly higher than the undoped graphite. These DFT calculation results reveal the mechanism of improved methanol adsorption on lithium-doped porous carbon and are consistent with the previous conclusions.

## 4. Conclusions

In summary, porous carbon with a high specific surface area and large pore volume was prepared using benzimidazole as a carbon precursor and KOH as an activating agent. Then, lithium doping was employed to improve the surface polarity of the porous carbon and enhance its methanol adsorption performance. The results showed that lithium doping could increase the micropore volume and decrease the mesopore size of the porous carbon, resulting in a methanol adsorption capacity of 35.4 mmol g^−1^, which was 57% higher than that of the undoped porous carbon. Molecular simulation results indicated that lithium doping could lower the threshold pressure of methanol adsorption, mainly due to the increased polarity of the carbon framework caused by lithium doping, which could form strong interactions with the OH groups in methanol. At 15 kPa, compared to pure graphite (2 nm), the pore size of the filled adsorption in the lithium-doped narrow pore model increased to 3 nm, and the adsorption capacity of the 4–6 nm pores also increased. DFT calculations revealed that the adsorption energy of lithium-modified graphite for methanol (−90.27 kJ mol^−1^) was much higher than that of unmodified graphite; the adsorption distance between lithium atoms and methanol molecules was 1.91 Å, lower than the distance between hydrogen and oxygen in graphite (2.41 Å). Additionally, lithium-doped graphite transferred 0.33 *e* of electrons to the oxygen atoms in methanol, significantly higher than the undoped case (0.03 *e*). The mechanism of methanol adsorption by lithium groups was revealed through experimental and theoretical calculations, providing a theoretical basis for the design and preparation of methanol adsorbents. In addition, Li-doped porous carbon can improve the surface polarity of the carbon surface, and can improve the adsorption and separation performance of CO_2_ and the separation of light hydrocarbon gases.

## Figures and Tables

**Table 1:** Lennard-Jones parameters used in calculation of force field.

Atom	ε (K)	σ (Å)	*q* (e)
C (acetone)	40	3.82	0.424
O (acetone)	79	3.05	−0.424
CH_3_ (acetone)	98	3.75	0.000
H (methanol)	-	-	0.435
O (methanol)	93	3.02	−0.700
CH_3_ (methanol)	98	3.75	0.265
C (surface)	47.86	3.47	Calc.
O (surface)	48.16	3.03	Calc.
H (surface)	7.65	2.85	Calc.
Li (surface)	12.58	2.45	Calc.

**Table 2:** Textural properties and surface chemistry of PC samples.

Sample	*S*_BET_/m^2^ g^−1^	*V*_t_/cm^3^ g^−1^	*V*_micro_/cm^3^ g^−1^	*V*_mes_ + *V*_mar_/cm^3^ g^−1^	O/at. %	N/at. %	Li/at. %
PC	2268	1.716	0.406	1.31	9.35	1.64	0
LiPC-1	2511	1.664	0.489	1.37	13.35	1.47	8.8
LiPC-2	2300	1.508	0.443	1.065	16.39	1.43	11.04
LiPC-3	2678	1.688	0.535	1.153	11.67	1.42	12.99

**Table 3:** Equilibrium amount of methanol adsorbed on various adsorbents.

Adsorbents	*Q*_e_ (mmol g^−1^)	Temperature (°C)	Pressure or Inlet Concentration	References
AC (YP-50)	16.6	25	15 kPa	[30]
CN950	13.2	25	15 kPa	[29]
A5	3.8	25	16 kPa	[35]
HPCMF.	7.6	25	15 kPa	[36]
PAF-12	9.0	25	16 kPa	[37]
OMP	13.7	25	15 kPa	[38]
PAF-5	29.2	25	16 kPa	[31]
PAF-20	19.0	25	16 kPa	[32]
PAF-11	20.4	25	16 kPa	[39]
AC/ZnO composite	15.0	25	200 g m^−3^	[34]
AC/MgO composite	12.3	25	200 g m^−3^	[34]
AC/CuO composite	11.0	25	200 g m^−3^	[34]
AC/ZrO composite	13.9	25	200 g m^−3^	[34]
HKUST-1	17.8	25	17.5 kPa	[8]
MIL-101Cr	35.9	25	13 kPa	[8]
Cu-BTC	20.1	25	12 kPa	[33]
LiPC-3	35.4	25	15 kPa	Present work

## Supplementary Materials

The following supporting information can be downloaded at: https://www.mdpi.com/article/10.3390/nano13182564/s1. References [42,43,44,45,46,47] are cited in the Supplementary Materials.
