# Supplementary material for: Revealing the Adsorption Mechanisms of Methanol on Lithium-Doped Porous Carbon through Experimental and Theoretical Calculations
Source: Nanomaterials (Basel). 2023 Sep 15;13(18):2564. doi: 10.3390/nano13182564 (PMC10537878; doi:10.3390/nano13182564)
Supplement: Supplementary file 1 [file nanomaterials-13-02564-s001.zip › nanomaterials-2597602-supplementary.pdf]

Revealing the Adsorption Mechanism of Methanol on Lithium-doped Porous Carbon through Experimental and Theoretical Calculations

Yiting Luo[1], Fangmu Aoer[2], Hanqing Wang[3], Xiangrong Dai[4], Rongkui Su[3,4,*] and Xiancheng Ma[2,*]

[1] Hunan First Normal University, Changsha 410114, China

[2] College of Mechanical and Electrical Engineering, Central South University of Forestry and Technology, Changsha, 410004, PR China

[3] College of Environmental Science and Engineering, Central South University of Forestry and Technology, Changsha, 410004, PR China

[4] PowerChina Zhongnan Engineering Corporation Limited, Changsha 410004, China;

*To whom correspondence should be addressed. Phone: 86-731-85623372; fax: +86-731-85623372; Rongkui Su. e-mail address: surongkui@csuft.edu.cn; Xiancheng Ma. e–mail address: xianchengma@hotmail.com;

List of Contents

Text S1 Material characterization and computational methodology R1

References R5

Test S1 Material characterization and computational methodology

1.1 Material characterization

The experimental samples were analyzed for their appearance and dimensions using a scanning electron microscope (SEM) model JSM-6700-F from JEOL Ltd., Japan. X-ray diffraction analysis was performed on the samples using a German Bruker D8 Advancel X-ray diffractometer with graphite monochromator and Cu/Kα radiation. The crystal structure analysis was conducted in the scanning range of 5° to 80° at a scanning speed of 10°/min. The specific surface area and gas adsorption of the samples were measured using a JW-BK132Z surface area and gas adsorption analyzer (Beijing JW-BK132Z Technology Co., Ltd., China) at -196°C. The total pore volume was calculated at a relative pressure of 0.995, while the specific surface area was determined using the Brunauer-Emmett-Teller (BET) method at relative pressures of 0.05 to 0.25. The pore size distribution (PSD) was calculated using the non-local density functional theory (NLDFT). X-ray photoelectron spectroscopy (XPS) analysis was performed using a K-Alpha 1063 instrument to investigate the surface elemental composition and bonding configurations. Methanol sorption isotherms were obtained using a JW-BK132Z instrument (Beijing JWGB Sci & Tech Co., Ltd) at 25 ℃. Prior to the adsorption analysis, the sample (around 50 mg) was degassed at 150 ℃ for several hours. After adsorption isotherm, the sample was degassed at 150 ℃ for 4 h under vacuum condition. Then, this sample was tested again for methanol adsorption.

1.2 Density functional theory (DFT)

All of the first principle calculations were conducted on the basis of density functional theory (DFT) with the use of the $DMol^3$ code [1] . The energy of the functional group and the interaction of carbon dioxide was calculated using the DFT calculation coupled with the van der Waals correct correction (DFT-D) [2] . Perdew,

Burke, and Ernzerhof (PBE) within the generalized gradient approximation (GGA-PBE) was selected [3] . The atomic orbit is described using double numeric polarization (DNP) basis set, which is comparable to 6-31G (d, p). The type of core processing is set up using a DFT half-core Pseudopots (DSPP) specifically designed for DMol3 calculations [4] . The real-space orbital global cutoff is 3.7 Å. The convergence threshold parameters for the optimization were 10^{-5} Hartree (energy), 2×10^{-3} Hartree (gradient), and 5×10^{-3} Hartree (displacement), respectively [5] . Therefore, BSSE effects need not be taken into consideration for calculating the energies and Mulliken charge. The methanol adsorption energy is calculated in the following manner:

$$E_{\text{ads}} = E_{\text{surface+methanol}} - (E_{\text{surface}} + E_{\text{methanol}}) \tag{S1}$$

where E_{ads}, $E_{\text{surface+ methanol}}$, and E_{methanol} are adsorption energy and total energy of adsorption-adsorbate complex, carbon surface, and isolated methanol, respectively. The binding energy of methanol in group-functionalized surface is a significant parameter for the methanol adsorption on the group-functionalized surface. An enhancement in the E_{ads} would be highly beneficial to the porous carbon for capturing methanol.

1.3. Grand canonical Monte Carlo (GCMC)

The following three parameters: pore size, the type and amounts of functional groups are investigated in slit-like pore model. Simulations are performed using Materials Studio 8.0 software. The initial structures of slit-pore graphitic were subject to geometric optimization within the DFT calculation. Perdew, Burke, and Ernzerhof (PBE) within the generalized gradient approximation (GGA-PBE) was selected [3] . The atomic orbit is described using double numeric polarization (DNP) basis set, which is comparable to 6-31G (d, p). The type of core processing is set up using a DFT half-core Pseudopots (DSPP) specifically designed for $DMol^3$ calculations [4] . The real-space orbital global cutoff is 3.7 Å. The convergence threshold parameters for the

optimization were 10^{-5} Hartree (energy), 2×10^{-3} Hartree (gradient), and 5×10^{-3} Hartree (displacement), respectively [5] . Charge partitioning was achieved through the mulliken method, and core-level charge was included in the integrations.

The hierarchical carbon-based pore structures were approximated as a collection of independent, functionalized graphitic slit-pores (**Figure S1**). The experimentally determined PSD was partitioned such that the full structure could be approximated as a weighted sum of pores with the following internal diameter 0.6, 0.7, 0.8, 1.0, 1.2, 1.5, 2.0, 3.0, 4.0 and 6.0 nm, where the pore width was assumed as the distance between carbon atoms at opposing pore walls less the collision diameter of a surface carbon atom (3.35 Å). Pores were created by first transposing the functional group from the center of the cell (where optimized) to the side, then mirroring the GGA-PBE-optimized cells about a point of inversion at the pore center. Functional groups were transposed to avoid unnatural overlap in the smaller pore sizes. The systems investigated include the perfect graphite basal plane surface, and functionalized groups. Grand canonical Monte Carlo (GCMC) simulations were carried out to describe acetone and methanol adsorption on the idealized functional pores described above [6] . The L-J potential parameters and partial charges of the methanol and acetone adsorbates were obtained from TraPPE-UA (united atom) force field.

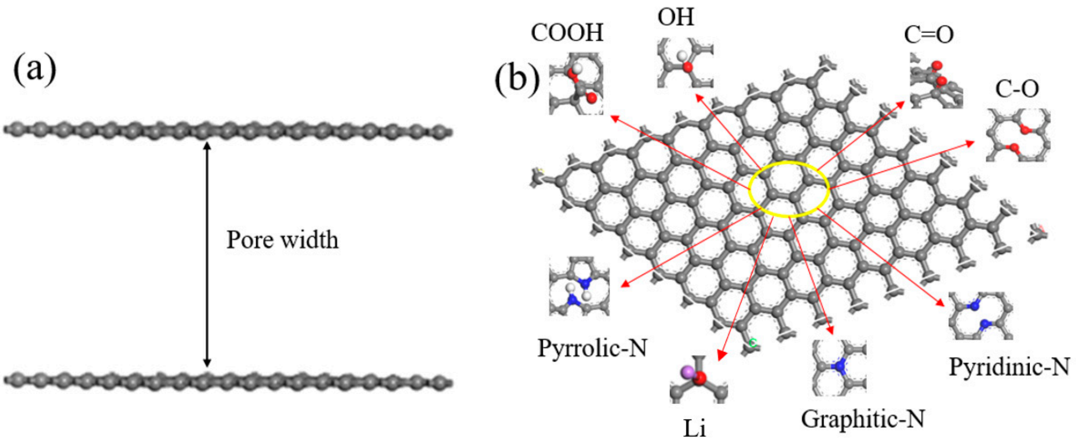

Figure S1 (a) Model of slit-shaped pore, and (b) nitrogen and oxygen modified graphite surfaces.

References

[1] Yang L., Jing L., Ming C. and Zheng C., Theoretical studies of CO_2 adsorption mechanism on linkers of metal–organic frameworks, Fuel, 2012, 95(1): 521–527.

[2] Grimme S., Semiempirical GGA-type density functional constructed with a long-range dispersion correction, Journal of Computational Chemistry, 2006, 27(15): 1787-1799.

[3] Lim G., Lee K. B. and Ham H. C., Effect of N-Containing Functional Groups on CO_2 Adsorption of Carbonaceous Materials: A Density Functional Theory Approach, Journal of Physical Chemistry C, 2016, 120(15): 8087-8095.

[4] Wang X., Liu Y., Ma X., Das S. K., Ostwal M., Gadwal I., Yao K., Dong X., Han Y. and Pinnau I., Soluble Polymers with Intrinsic Porosity for Flue Gas Purification and Natural Gas Upgrading, Advanced Materials, 2017, 29(10): 1605826.

[5] Liu Y., Liu J., Chang M. and Zheng C., Effect of Functionalized Linker on CO_2 Binding in Zeolitic Imidazolate Frameworks: Density Functional Theory Study, Journal of Physical Chemistry C, 2012, 116(32): 16985-16991.

[6] Gupta A., Chempath S., Sanborn M. J., Clark L. A. and Snurr R. Q., Object-oriented programming paradigms for molecular modeling, Molecular Simulation, 2003, 29(1): 29-46.
